# Supplementary material for: Mn2+-activated dual-wavelength emitting materials toward wearable optical fibre temperature sensor
Source: Nat Commun. 2022 Apr 20;13:2166. doi: 10.1038/s41467-022-29881-6 (PMC9021195; doi:10.1038/s41467-022-29881-6)
Supplement: Supplementary file 1 — Supplementary Information [file 41467_2022_29881_MOESM1_ESM.pdf]

Mn^{2+}-activated dual-wavelength emitting materials toward wearable optical fibre temperature sensor

Song et al.

Contents

Cytotoxicity measurement 1
Supplementary Table 1..2
Supplementary Figure 1...3
Supplementary Figure 2...4
Supplementary Figure 3...5
Supplementary Figure 4...6
Supplementary Figure 5...7
Supplementary Figure 6...8
Supplementary Figure 7...9
Supplementary Figure 8...10
Supplementary Figure 9...11
Supplementary Figure 10...12
Supplementary Figure 11...13
Supplementary Figure 12...14

Cytotoxicity measurement.

MTT assays were used to evaluate the cytotoxicity of fiber. The fibre was soaked in 3 mL DMEM solution containing 1% penicillin-streptomycin and 10% FBS at 37 ℃, then 1 mL of leaching solution was taken out at 0, 6, 12, 24 h. The cells were seeded in a 96-well plate (Costar, IL, USA) with a density of 1×10^5 cells/mL. After incubation for 24 h, medium in each well were replaced by 100 μL fibre leaching solution. After further incubation for 24 h, the medium was removed and fresh-prepared MTT solution (0.5 mg/mL, 100 μL) was added. After incubation at 37 ℃ for 4 h, the MTT solution was removed and DMSO (100 μL) was then added into each well and the plate was gently shaken for 10 min at room temperature to dissolve all the precipitates. The absorbance of sample and control wells at 570 nm were then measured by a microplate reader. Cell viability was then calculated by the ratio of the absorbance of sample wells to control cells.

Supplementary Table 1. The fitting parameters of the Mn K-edge EXAFS cures in $Li_2Zn_{1-x}SiO_4$:$x$$Mn^{2+}$ (x = 0.05, 0.15). CN (coordination Number), ΔE_0 (Edge-energy shift), σ^2(Debye Waller factor).

Sample	Path	N	ΔE(eV)	100×R(Å)	$10^3\times\sigma^2$(Å^2)	R-factor
x = 0.05	Mn-O	4.49(0.47)	4.89(1.39)	203.3(1.1)	2.80(1.51)	0.008
x = 0.15	Mn-O	3.36(0.81)	8.27(2.16)	194.3(1.7)	4.04(2.09)	0.006
	Mn-Mn	2.33(0.64)	8.27(2.16)	218.6(3.0)	4.04(2.09)	

For x = 0.05, the fitting ranges: k space: 3.0≤k≤11.6; R space: 1≤R≤2.2. For x = 0.15, the fitting ranges: k space, 3.2≤k≤11.2; R space: 1≤R≤3.4.

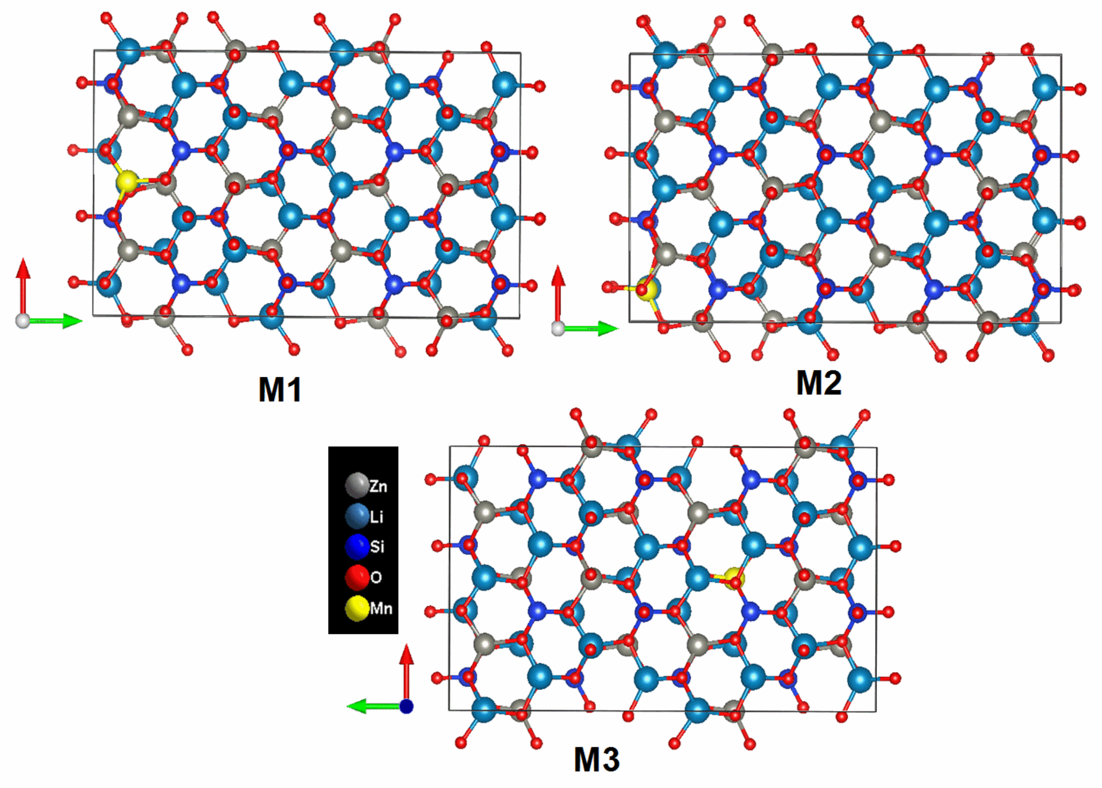

Supplementary Figure 1. Substitution models of a 2×2×2 Li_2ZnSiO_4 supercell with one cation replaced by one Mn^{2+} after structure optimization. (M1: $Mn_{Li1}^{\bullet} + V_{Li1}^{'}$; M2: $Mn_{Li2}^{\bullet} + V_{Li2}^{'}$; M3: $Mn_{Zn}^{\times}$)

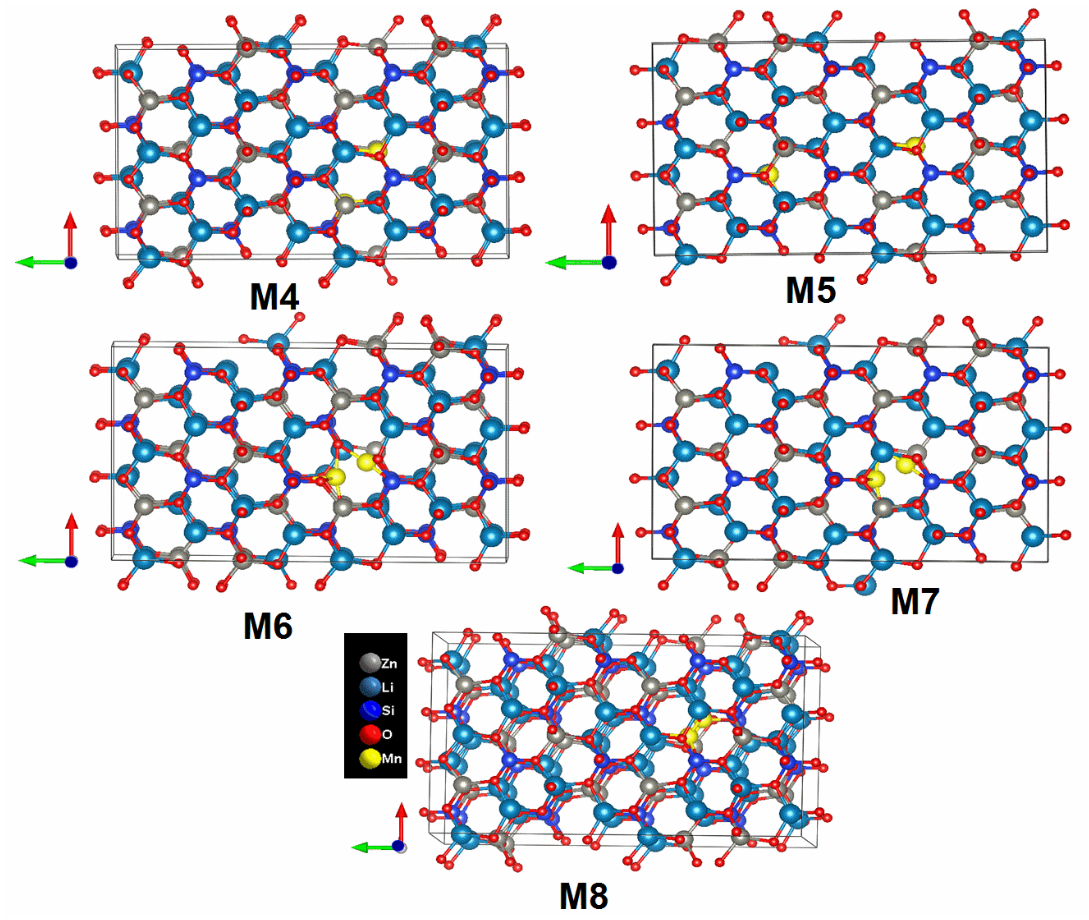

Supplementary Figure 2. Substitution models of a 2×2×2 Li_2ZnSiO_4 supercell with two cations replaced by two Mn^{2+} ions after structure optimization.

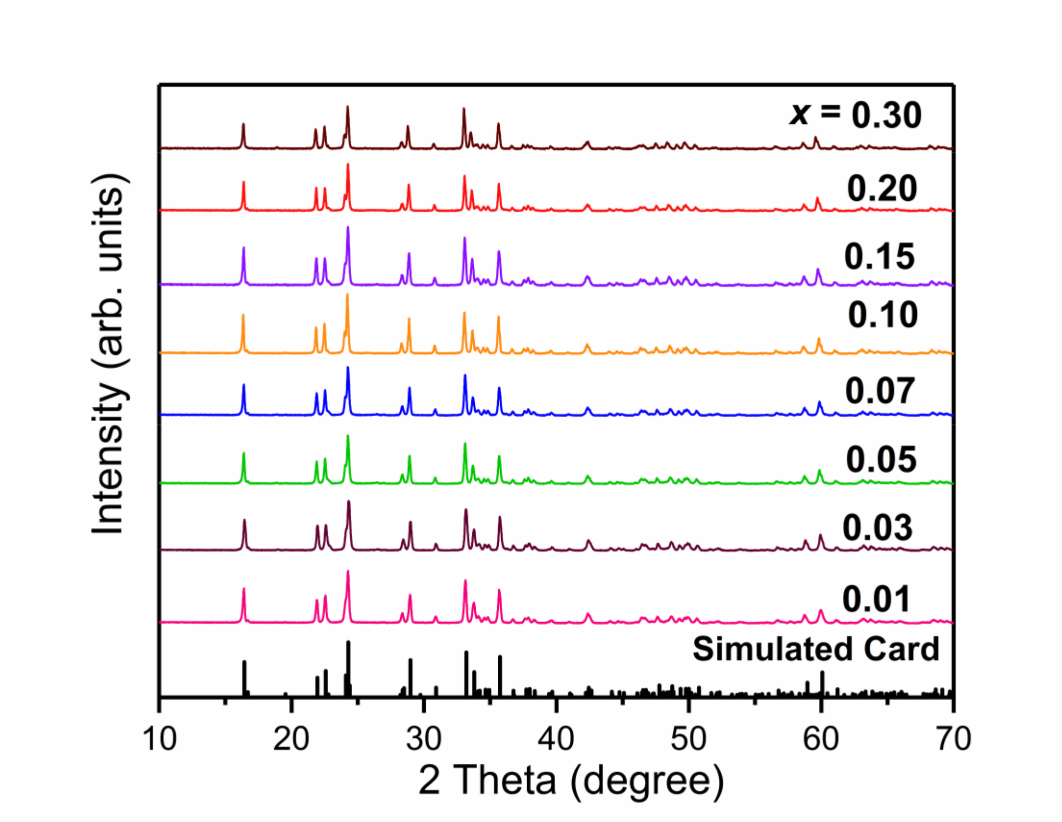

Supplementary Figure 3. XRD patterns of $Li_2Zn_{1-x}SiO_4$:$x$$Mn^{2+}$ (x = 0.01-0.30) samples. The simulated card of Li_2ZnSiO_4 is given for comparison.

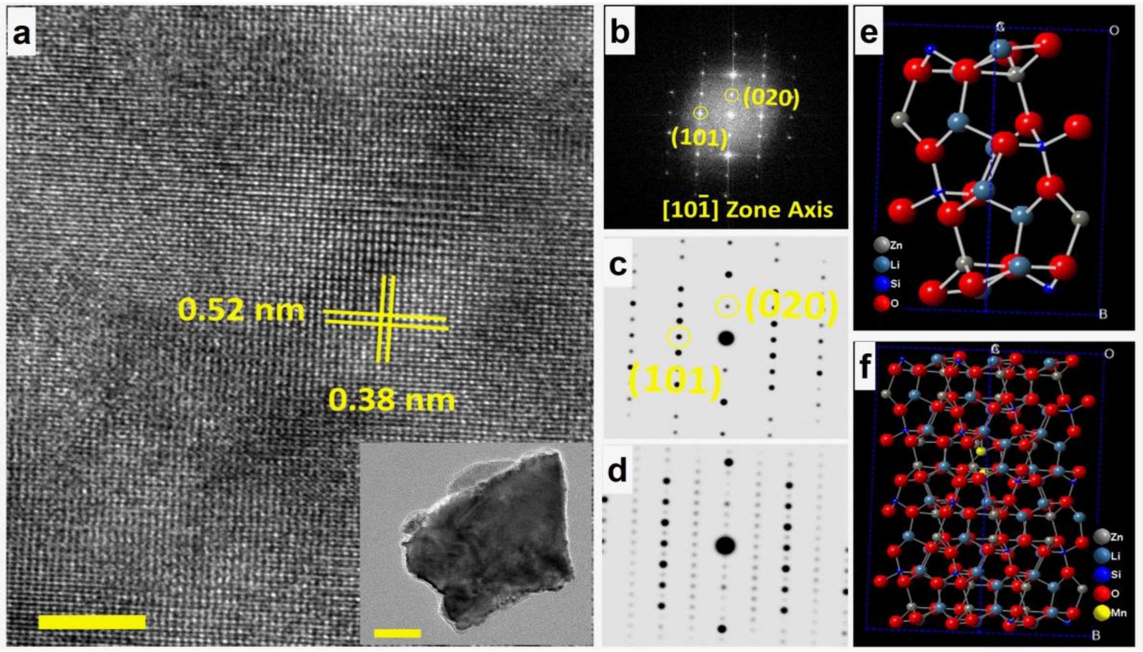

Supplementary Figure 4. a HR-TEM image of a $Li_2Zn_{0.95}SiO_4$:0.05Mn^{2+} particle. Scale bar: 5 nm. The inset shows the low-resolution TEM image of the $Li_2Zn_{0.95}SiO_4$:0.05Mn^{2+} particle. Scale bar: 50 nm. **b** Fourier-transform diffraction patterns of the $Li_2Zn_{0.95}SiO_4$:0.05Mn^{2+} particle viewed along $[10\bar{1}]$ zone axis taken from the HR-TEM in a). **c,e** Simulated electron diffraction pattern and the corresponding crystallographic model of Li_2ZnSiO_4 viewed along $[10\bar{1}]$ zone axis. **d,f** The simulated electron diffraction pattern and the corresponding crystallographic model of M7 viewed along $[10\bar{1}]$ zone axis**.**

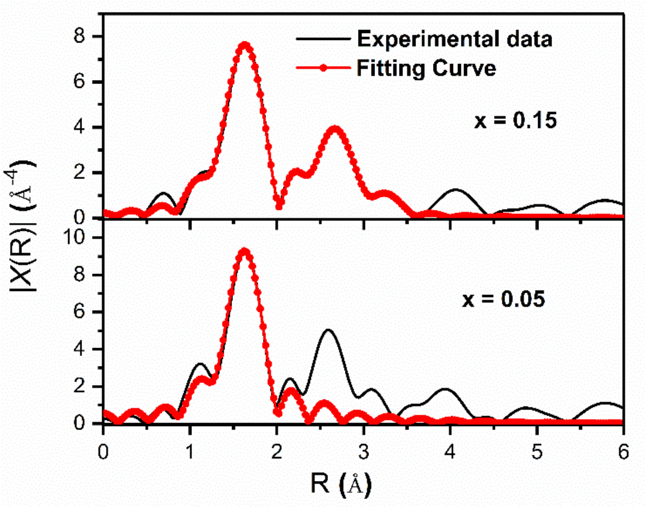

Supplementary Figure 5. The EXAFS experimental data and fitting curves in R-space of $Li_2Zn_{1-x}SiO_4$:$x$$Mn^{2+}$ (x = 0.05, 0.15). A multi-shell fit model was applied to determine the local structure of Mn^{2+} in the two samples, and an R-factor of 0.006–0.008 was obtained for the fittings, suggesting that the fitting results are good and credible. The detailed fitting parameters are summarized in Supplementary Table 1. The coordination number (CN) for Mn^{2+} is 4.49 ± 0.47, which is close to 4 in $Li_2Zn_{0.95}SiO_4$:0.05Mn^{2+}, and the Mn-O distance is ~2.033 Å, indicating that Mn^{2+} occupied four coordinated Zn sites in this sample. For the $Li_2Zn_{0.85}SiO_4$:0.15Mn^{2+} sample, the fitted Mn–O distance is ~1.943 Å and the CN is 3.36 ± 0.81, which is slightly smaller than 4, suggesting that the Mn^{2+} ions simultaneously occupy both the three coordinated Li2 and four coordinated Zn sites in this sample. Moreover, the fitting results also showed that Mn^{2+} ions existed in the second shell of Mn^{2+}, and the Mn^{2+}-Mn^{2+} distance is ~2.186 Å, which is closer to the shortest Zn^{2+}–Li^{+} distance (~3.164 Å) than the minimal Zn^{2+}–Zn^{2+} distance (4.386 Å) in Li_2ZnSiO_4. Besides, it is also close to the calculated Mn^{2+}(Zn)–Mn^{2+}(Li2) distance (1.87 Å). Therefore, it is concluded that the Mn^{2+} ions occupied Zn and Li2 sites in $Li_2Zn_{0.85}SiO_4$:0.15Mn^{2+}. Additionally, Mn^{2+}(Zn) and Mn^{2+}(Li2) would form the dimer by sharing with one oxygen, and the new emission band from the Mn^{2+}-Mn^{2+} dimer in addition to the isolated Mn^{2+} emission can be expected in the samples doped with relatively high concentrations of Mn^{2+}.

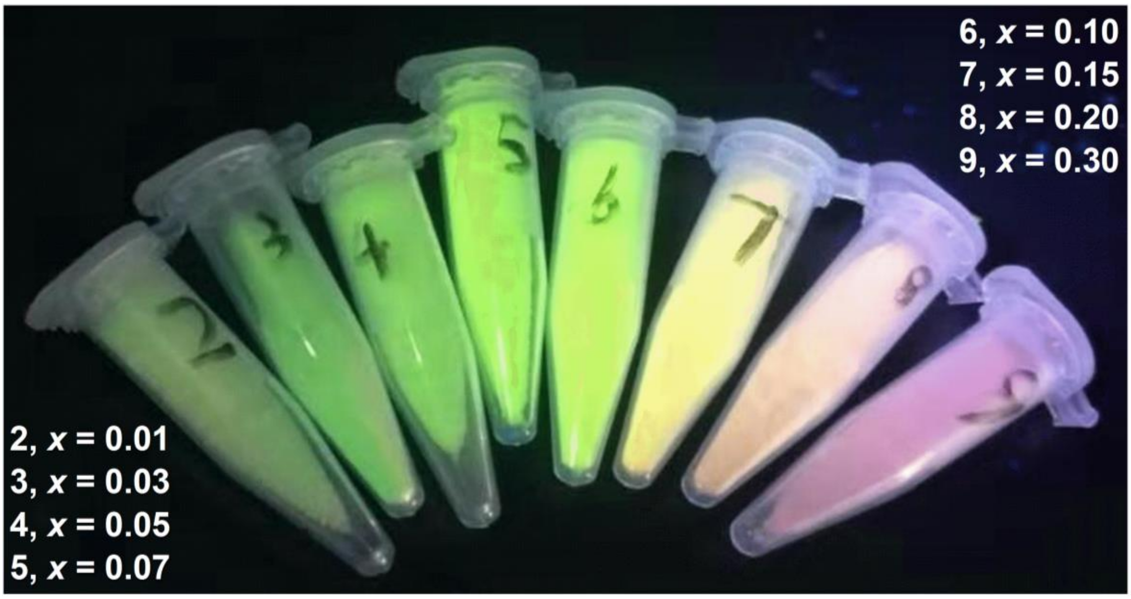

Supplementary Figure 6. luminescence images of the phosphor samples $Li_2Zn_{1-x}SiO_4$:$x$$Mn^{2+}$ (x = 0.01-0.30) upon 365 nm UV-light excitation.

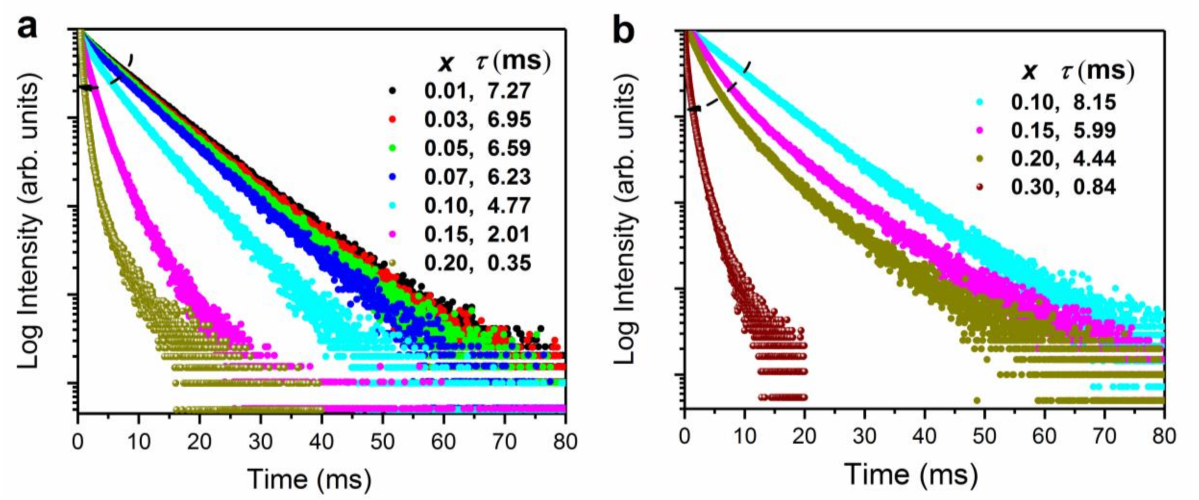

Supplementary Figure 7. Luminescence decay curves of the green (a) and red (b) emission bands in $Li_2Zn_{1-x}SiO_4$:$x$$Mn^{2+}$ (x= 0.01-0.30).

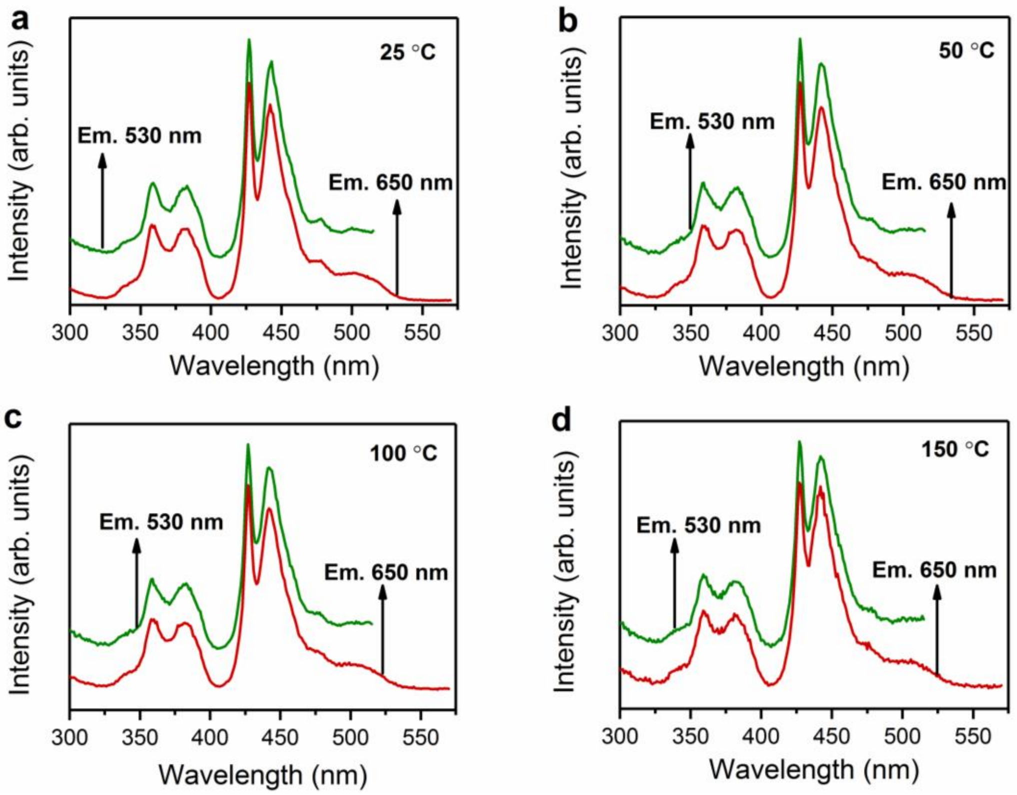

Supplementary Figure 8. a-d The excitation spectra of green and red emissions in $Li_2Zn_{0.85}SiO_4$:0.15Mn^{2+} at different measure temperatures ranging from 25–150 ℃.

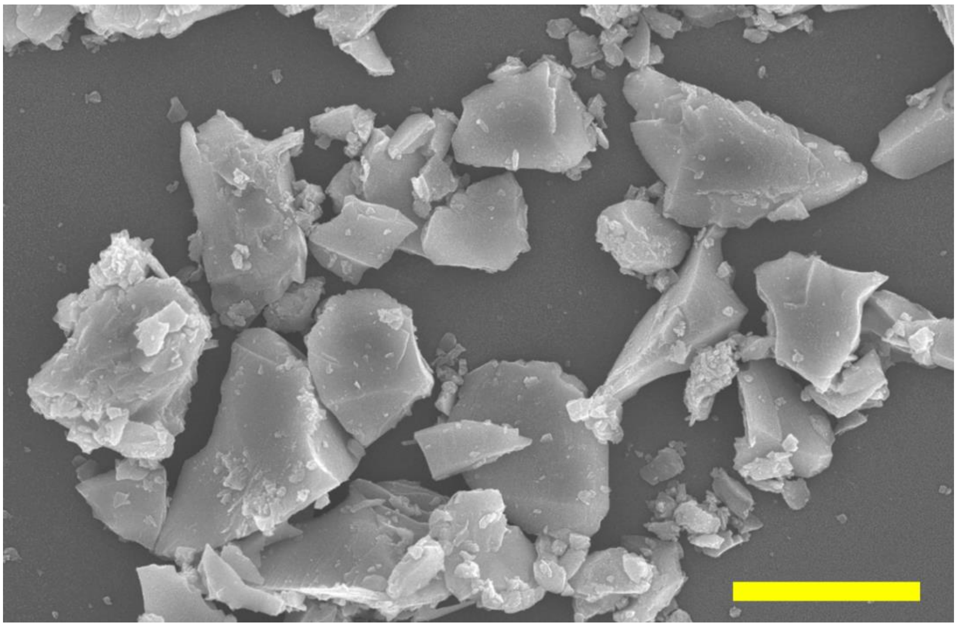

Supplementary Figure 9. SEM image of $Li_2Zn_{0.85}O_4$:0.15Mn^{2+}. Scale bar: 5 μm.

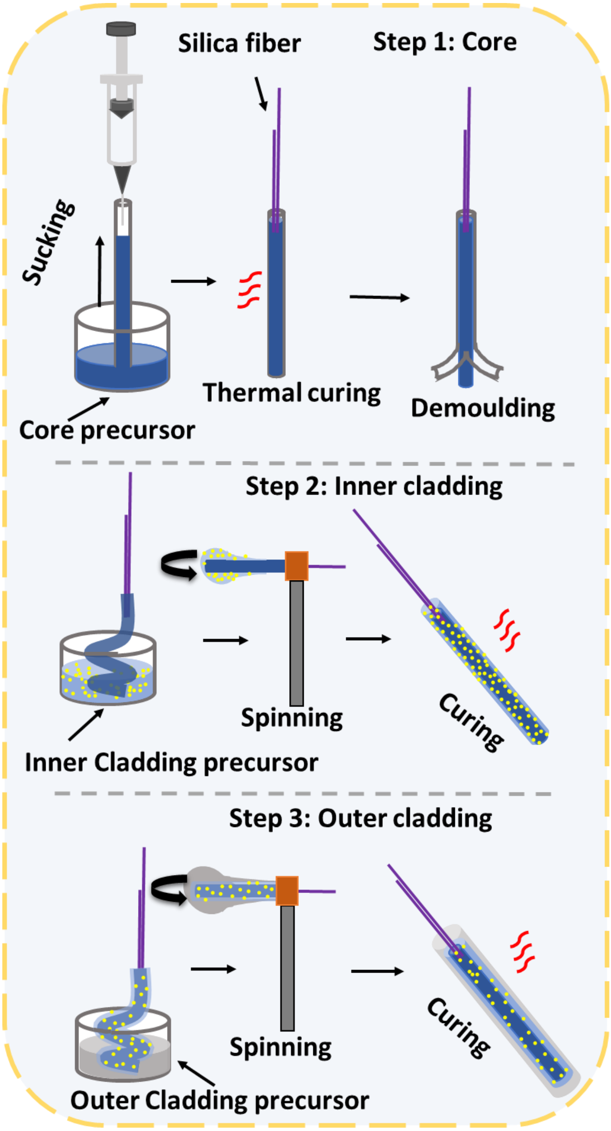

Supplementary Figure 10. Schematic illustration of the fabrication procedure of the optical fibre. First, the base and curing agent of OE with a quality ratio of 1:1 was chosen as the core precursor solution. After stirring and degassing, the core precursor solution was sucked into a Teflon tube with a syringe, and the diameter of the core was determined by the size of tube. Afterwards, a section of two 200/400 μm silica optical fibres was inserted into the tube as a pigtail. After thermal curing was performed at 100 ℃ for 2 h, the fibre core was obtained by peeling off the tube with scissors and tweezers. Subsequently, the fluorescence response layer was coated by dipping the fibre core into the configured inner cladding precursor solution, which is a mixture of the OE precursor solution and $Li_2Zn_{0.85}SiO_4$:0.15Mn^{2+} powder (quality

ratio of 10:1). Next, the coated core was spun horizontally using a rotating motor at 2000 rpm for 2 min. Thereafter, it was thermally cured at 100 ℃ for 2 h to form a homogeneous inner cladding layer. The outer cladding of the fibre was prepared by a similar method, and it was thermally cured at 90 ℃ for 40 min, while the precursor was the mixture of the base and curing agent of PDMS (quality ratio of 10:1). The thickness of the cladding was controlled by the speed and spinning time of the motor. The numerical aperture ($NA = \sqrt{n_1^2 - n_2^2}$) of the designed optical fibre is about 0.59, exhibiting a good ability to collect and guide light.

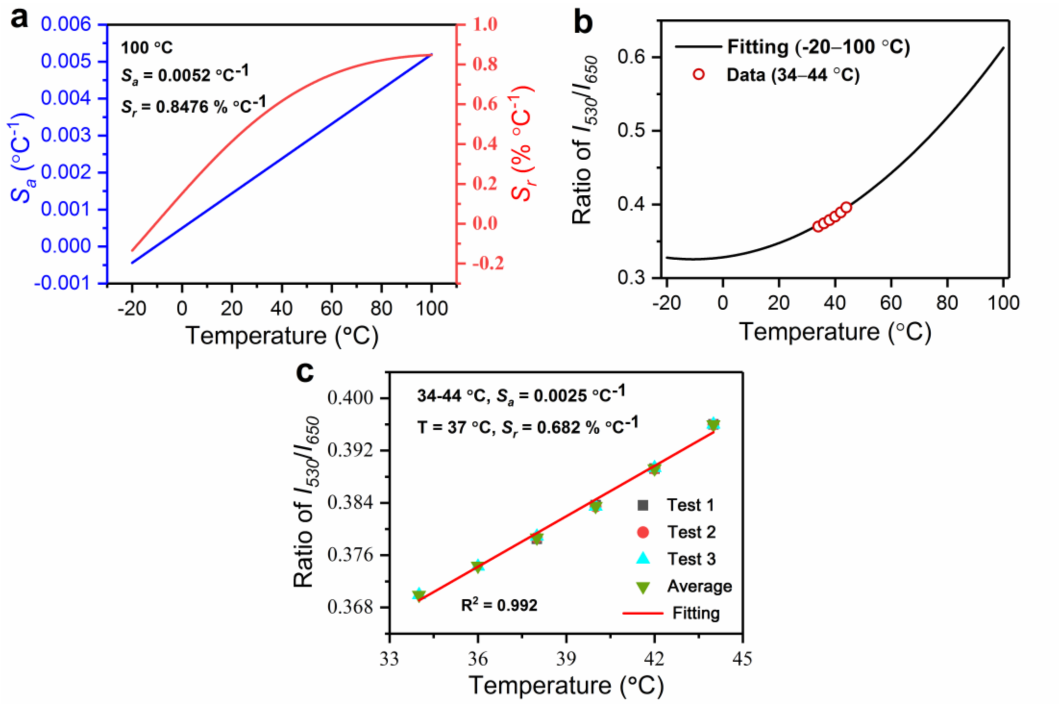

Supplementary Figure 11. a The S_a and S_r values of the temperature sensor as a function of the measure temperature according to Fig. 5b. **b** The fitting curve of the emission ratios of green (530 nm) to red emission (650 nm) based on Fig. 5b and the measured emission ratios of green (530 nm) to red emission (650 nm) with an interval of 2 ℃ (red circle). **c** The linear fitting curve (y = 0.00257T+0.2817) of the emission ratio of the green (530 nm) to red emission (650 nm) and the corresponding experimental data according to Supplementary Figure 11b (red circles).

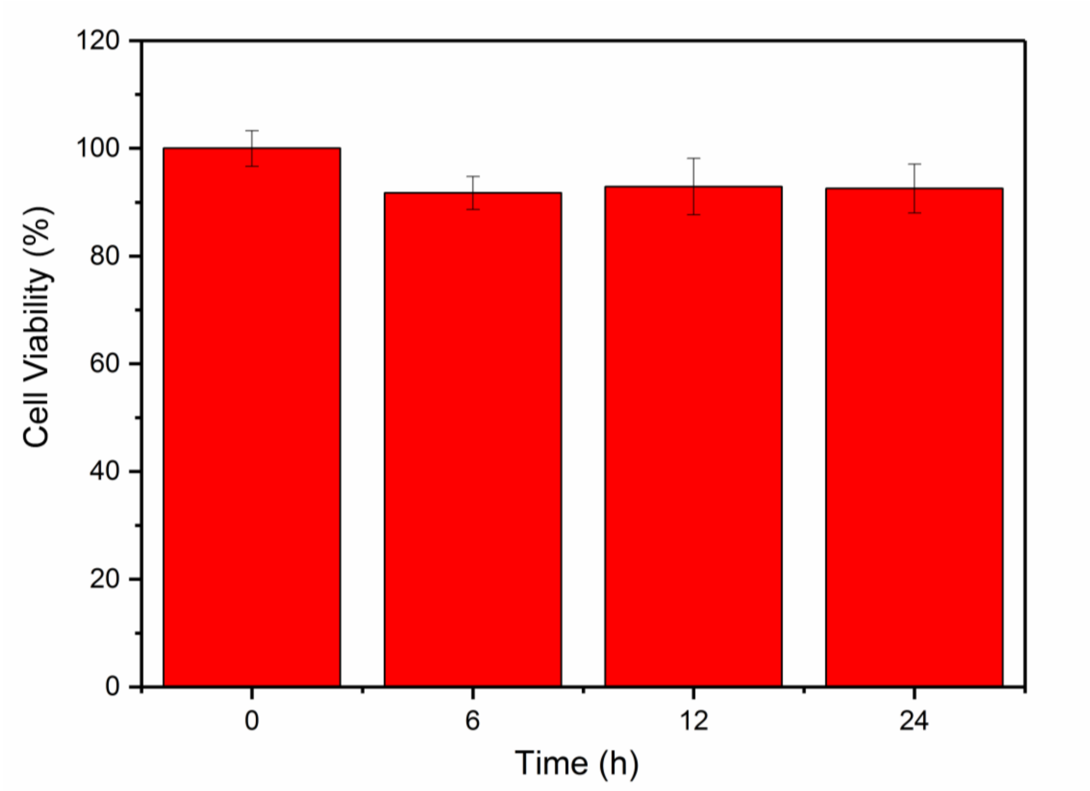

Supplementary Figure 12. Cell viability as a function of the time in a culture medium (contained the fabricated fibre).
